# Clinical and Epidemiological Features of Hospitalized Dengue Cases During the 2025 Outbreak in Mymensingh, Bangladesh

**DOI:** 10.7759/cureus.109609

**Published:** 2026-05-25

**Authors:** Md. Mohiuddin Khan, Md. Rasel, Md. Khurshed Alam, Foysal Ahamed, Md. Khairul Islam, A. H. M. Shalakin Mamun, Zyed Md. Adnan Shameem, Mahmud Anik, Tanzin Naher, Jannatul Fardous

**Affiliations:** 1 Department of Medicine, Mymensingh Medical College Hospital, Mymensingh, BGD; 2 Department of Public Health, Tropical Disease and Health Research Center, Dhaka, BGD

**Keywords:** 2025, bangladesh, dengue, epidemiology, geographical expansion, mymensingh

## Abstract

Background: Dengue fever is a significant public health challenge both globally and in Bangladesh due to the increasing disease burden and expanding geographic distribution. This study aimed to explore the clinical spectrum, disease severity distribution, and in-hospital outcomes of hospitalized adult dengue patients in Mymensingh, Bangladesh, during the 2025 outbreak, within a regional context.

Methods: This study was a hospital-based prospective observational study conducted at Mymensingh Medical College Hospital (MMCH), Mymensingh, Bangladesh, from July to October 2025. About 380 adult patients admitted with confirmed dengue infection were included. Acute dengue virus infection was confirmed using laboratory criteria, defined as a positive serological test for dengue nonstructural protein 1 (NS1) antigen or dengue-specific IgM/IgG antibodies via rapid immunochromatographic assay or enzyme-linked immunosorbent assay (ELISA). Demographic data were collected through face-to-face interviews. Clinical findings on the day of admission were collected through physical examination and laboratory investigations. In-hospital outcome was also recorded. Disease severity was assessed according to the revised WHO 2009 dengue case classification. Collected data was analyzed using IBM SPSS Statistics, version 23.0 (IBM Corp., Armonk, NY, USA).

Results: The mean age of the study population was 31.3 ± 11.1 (SD) years, with male predominance (315 (82.9%)). The majority of patients resided in urban areas (310 (82.1%)), and 308 (81.1%) reported a recent history of travel. Common clinical manifestations included fever and arthralgia (380 (100%) each), headache (379 (99.7%)), myalgia (357 (93.9%)), cough (250 (65.8%)), and nausea (224 (58.9%)). Warning signs were observed in 235 (62.2%) cases, while severe dengue was reported in five (1.3%). Mortality was reported in two (0.5%) cases, and four cases were referred to a higher facility.

Conclusion: Young adult males constituted the majority of hospitalized dengue patients in this regional outbreak. While a high proportion presented with warning signs, hospitalization and management resulted in a low rate of progression to severe dengue and minimal mortality. These findings point towards an expanding geographical footprint of dengue beyond classical metropolitan hotspots and emphasize the critical need for decentralized healthcare preparedness, regional vector control, and mobility-linked surveillance systems across Bangladesh.

## Introduction

Bangladesh has been facing a series of intense dengue outbreaks over the last two decades, with a concerning increase in cases and disease severity [1]. It is a mosquito-borne hemorrhagic disease caused by the dengue virus, an arbovirus of the *Flavivirus *genus, transmitted primarily by *Aedes aegypti* and *Aedes albopictus* mosquitoes [2]. Dengue fever has been observed with distinct epidemiological patterns in Bangladesh, with cases peaking during monsoon, typically from June to September, and post monsoon, typically from October to November [3]. Alongside this, a shift in virus serotype predominance has been observed, which has been aggravating disease severity and mortality [4].

In Bangladesh, dengue fever was initially confined mostly to the Dhaka division, particularly in urban areas [5]. Recently, the cases have expanded geographically, reaching other divisions including Chattogram, Khulna, and Mymensingh [5-8]. Mymensingh is situated in the tropical monsoon zone of Bangladesh, adjacent to the highly endemic Dhaka division. In recent years, this region has experienced a noticeable rise in reported dengue cases [9]. By October of 2025, over 1,000 hospitalizations have been reported due to dengue fever [10]. Heavy seasonal rainfall often leads to water stagnation in many parts of the city area and these waterlogged areas provide ideal breeding conditions for the *Aedes *mosquito vector. On a broader scale, global warming heavily exacerbates this local vulnerability by shifting rainfall patterns and elevating ambient temperatures across South Asia. These climate changes prolong the vector transmission season and shorten the viral incubation period. Consequently, the intersection of climate change and rapid urbanization is transforming dengue from a localized, seasonal outbreak into an expansive, year-round public health threat in regional hubs like Mymensingh. Close proximity to the endemic area of Dhaka facilitates the movement of people and the spread of the virus and vector. Hence, despite this rising local burden, epidemiological and clinical data from tertiary care centers in Mymensingh remain limited.

Mymensingh Medical College Hospital (MMCH) is a major tertiary-level government hospital that functions as a key referral center for both urban and rural populations across the Mymensingh division. This hospital faces a substantial clinical burden of dengue cases during the peak transmission season of 2025. Till 16^th^ November 2025, a total of 2,187 dengue cases were admitted in this hospital, with 18 reported deaths. Understanding the clinical presentations, demographic profiles, comorbidities, severity, and outcomes of hospitalized dengue patients within a specific local context is crucial. Therefore, this study aims to systematically document the clinical and epidemiological features of dengue patients admitted to this hospital.

The study findings serve to fill the knowledge gap regarding evolving epidemiology beyond the classical hotspot regions. Furthermore, this research informs healthcare providers and policymakers on resource allocation and clinical management strategies tailored to regional needs.

## Materials and methods

This prospective observational study was carried out at MMCH from July to October 2025 among hospital-admitted dengue patients. This hospital serves as a government-facilitated tertiary care hospital, situated in the Mymensingh division, a region characterized by a tropical monsoon climate and heavy seasonal rainfall.

Adult patients (≥18 years) admitted to MMCH from July to October 2025 with confirmed dengue fever (positive nonstructural protein 1 (NS1) antigen or dengue-specific IgM/IgG antibody tests) were considered for inclusion. Patients with concurrent febrile illnesses, including malaria, typhoid, or chikungunya, were excluded [11]. A total of 380 patients were finally enrolled following informed written consent.

After enrollment, detailed demographic and clinical histories of all patients were recorded. A thorough physical (both general and systemic) examination was conducted, and the findings were recorded. Anthropometric measures (height and weight) were taken. Laboratory investigations, including hematocrit, hemoglobin, white blood cell count, platelet count, and alanine aminotransferase (ALT), were conducted, and the reports were collected and recorded. These assessments were conducted on the day of admission. The in-hospital outcome of all these patients was also recorded. Data collection was conducted through a prestructured case record form to minimize interobserver variability (Appendix A).

The severity of dengue fever will be classified into three classes: dengue without warning signs, dengue with warning signs, and severe dengue following the WHO 2009 classification [12]. Here, abdominal pain, persistent vomiting, fluid accumulation, bleeding manifestations, lethargy, restlessness, hepatomegaly, and concurrent increase and decrease of hematocrit (>20%) and platelets, respectively, were considered as warning signs. Features of plasma leakage, severe bleeding, severe organ impairment, and significant metabolic imbalance were considered indicative of severe dengue. 

Body mass index (BMI) was calculated from height and weight using the standard formula: weight in kilograms divided by height in meters squared (kg/m²). Calculated BMI was categorized as underweight (<18.5), normal (18.5-22.9), overweight (23-24.9), and obese (≥25) [13]. Here, the cut-off value specified for the Asian population was considered.

Ethical considerations

The study was approved by the Institutional Review Board of Mymensingh Medical College (Memo no. MMC/IRB/2023/589). Written informed consent was obtained from all adult participants (≥ 18 years).

Statistical analysis

The data were analyzed via IBM SPSS Statistics, version 23.0 (IBM Corp., Armonk, NY, USA). Descriptive statistics were used to summarize the baseline characteristics of the study population. Continuous data were assessed for normality through the Shapiro-Wilk test, while data with normal distribution were reported as the means ± SDs, and data with non-parametric distribution were presented as median (minimum-maximum). Categorical data were presented as frequencies and percentages. Missing data were handled using a complete-case analysis approach, where percentages and frequencies for specific variables were calculated based on the total number of valid responses available for that metric. Missing values were excluded from the denominators of the respective analyses rather than being replaced through imputation. All adjusted denominators are explicitly stated across the text and tables to maintain transparency. We also acknowledge potential sources of bias, including recall bias in self‑reported travel and symptom history, which may affect the accuracy of certain exposures. 

## Results

The mean age of participants was 31.3 ± 11.1 (SD) years, and 315 (82.9%) were male. A large majority, 310 (82.1%), resided in urban areas, with an average monthly family income of 29887.1 ± 7277.6 Bangladeshi Taka. Regarding nutritional status, 44 (11.6%) were obese, and 110 (28.9%) were overweight, with a mean BMI of 21.8 ± 3.4 kg/m². Among comorbid conditions, 51 (13.4%) had diabetes mellitus and 38 (10%) had hypertension. Recent travel within the previous 30 days was reported by 308 (81.1%) of participants, most of whom traveled to areas within Dhaka, with nearly two-thirds (232, 61.1%) visiting locations in the Dhaka division (Table 1).

**Table 1:** Baseline characteristics of the study patients with dengue fever (N=380) BMI: body mass index Percentages were calculated as valid percentages excluding missing data for each variable. *Data are presented as mean ± standard deviation.

Characteristics of the patients	n (%)
Age group (years)	
18-30	232 (61.1)
31-40	87 (22.9)
41-50	34 (14.4)
51-60	20 (5.3)
61-70	7 (1.8)
Mean±SD*	31.3±11.1
Gender	
Male	315 (82.9)
Female	65 (17.1)
Residence (n=378)	
Urban	312 (82.1)
Rural	66 (17.4)
Monthly family income (Bangladesh Taka)*	29887.1±7277.6
BMI category	
Underweight	31 (8.2)
Normal	195 (51.3)
Overweight	110 (28.9)
Obese	44 (11.6)
Mean±SD (kg/m^2^) *	21.8±3.4
Comorbidities	
Diabetes mellitus	51 (13.4)
Hypertension	38 (10)
History of travelling in the last 30 days	308 (81.1)
Dhaka	232 (61.1)
Gazipur	57 (15)
Tangail	5 (1.3)
Narayanganj	2 (0.5)
Habiganj	1 (0.3)
Jamalpur	1 (0.3)
Kishoreganj	1 (0.3)
Madaripur	1 (0.3)

All 380 (100%) participants presented with fever and arthralgia, and nearly all had headache (379, 99.7%), and 357 (93.9%) had myalgia. The mean duration of fever on the day of admission was 7.02 ± 3.8 (SD) days, with the majority experiencing fever for four to six days.

General symptoms included red eyes (113, 29.7%), rash (37, 9.7%), and pallor (24, 6.3%). Features of plasma leakage (three, 0.8%), shock (one, 0.3%), and abdominal distension (one, 0.3%) were reported accordingly. Gastrointestinal features were reported as nausea (224, 58.9%), abdominal pain (155, 40.8%), ascites (114, 30.0%), and acute vomiting (102, 26.8%). Others included anorexia (49, 12.9%), diarrhea (33, 8.7%), persistent vomiting (20, 5.3%), hepatomegaly (25, 4.2%), and abdominal distension (one, 0.3%). Respiratory features were also reported, including cough (250, 65.8%), chest pain (99, 26.1%), pleural effusion (52, 13.7%), and respiratory distress (42, 11.1%). Bleeding manifestations included epistaxis (43, 11.3%), melena (24, 6.3%), gum bleeding (23, 6.1%), excessive menstrual bleeding (18, 4.7%), hemoptysis (17, 4.5%), hematemesis (eight, 2.1%), and hematuria (three, 0.8%). Regarding laboratory findings, the mean hematocrit, hemoglobin, and white blood cell count were 37.4 ± 5.3% (SD), 13.5 ± 1.59 g/dL (SD), and white blood cell count 7249.2 ± 7821.9 cells/µL (SD). The mean platelet count was 109000 ± 58299.9 cells/µL. Median ALT was 107.5 U/L (range: 25-852 U/L) (Table 2).

**Table 2:** Clinical presentations of the study participants with dengue fever on the day of admission (n=380) Data were presented as mean ± standard deviation* and median (minimum-maximum) **

Clinical presentations	n (%)	Reference range
General features		
Fever	380 (100)	-
Duration from onset of fever (days; n=372)		
≤ 3	39 (10.5)	-
4-6	167 (44.9)	-
≥ 7	166 (44.6)	-
Mean±SD	7.02±3.8	-
Arthralgia	380 (100)	-
Headache	379 (99.7)	-
Myalgia	357 (93.9)	-
Red eye	113 (29.7)	-
Rash	37 (9.7)	-
Pale	24 (6.3)	-
Restlessness	4 (1.1)	-
Palpitation	14 (3.7)	-
Edema	4 (1.1)	-
Loss of consciousness	2 (0.5)	-
Confusion	2 (0.5)	-
Dizziness	2 (0.5)	-
Features of plasma leakage	3 (0.8)	-
Shock	1 (0.3)	-
Gastrointestinal features		
Nausea	224 (58.9)	-
Abdominal pain	155 (40.8)	-
Ascites	114 (30)	-
Acute vomiting	102 (26.8)	-
Anorexia	49 (12.9)	-
Diarrhea	33 (8.7)	-
Persistent vomiting	20 (5.3)	-
Hepatomegaly	25 (4.2)	-
Abdominal distension	1 (.3)	-
Respiratory features		
Cough	250 (65.8)	-
Chest pain	99 (26.1)	-
Pleural effusion	52 (13.7)	-
Respiratory distress	42 (11.1)	-
Bleeding manifestations		
Epistaxis	43 (11.3)	-
Melena	24 (6.3)	-
Gum bleeding	23 (6.1)	-
Excessive menstrual bleeding	18 (4.7)	-
Hemoptysis	17 (4.5)	-
Hematemesis	8 (2.1)	-
Hematuria	3 (.8)	-
Laboratory findings		
Hematocrit (%) *	37.4±5.3	Males: 42-54%; Females: 38-46%
Hemoglobin (g/dl) *	13.5±1.59	Males 13.5-17.5 g/dL, Females 12-15.5 g/dL
White blood cell (cells/µL) *	7249.2±7821.93	4,000-11,000/µL
Platelet (cells/µL) *	109000±58299.9	150,000-450,000/µL
Alanine Aminotransferase (ALT) (U/L) **	107.5 (25-852)	10-40 U/L

**Figure 1:**
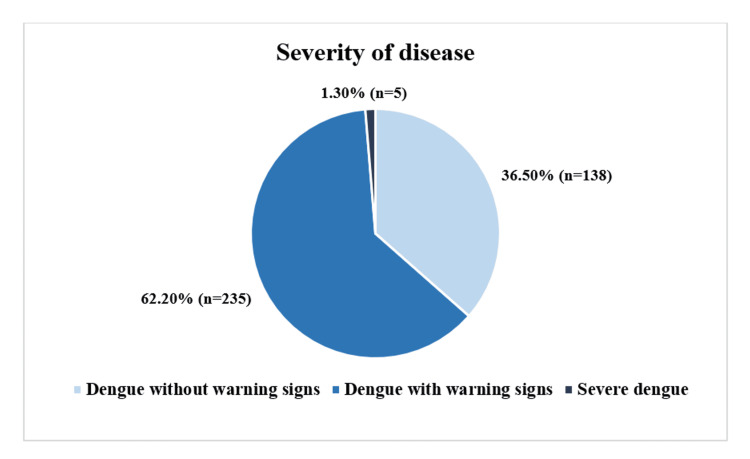
Severity of disease among study participants with dengue fever (n=378)

Three hundred and seventy-three (98.7% of all) patients were classified as having non-severe dengue, among whom 235 (62.2%) presented with warning signs and 138 (36.5%) had no warning signs. Five (1.3% of all) patients exhibited features consistent with severe dengue (Figure 1).

The in-hospital outcomes of the 374 study participants were recorded, while 361 (95%) were discharged with advice. About five (1.3%) were discharged on risk bond, and only three (0.8%) absconded. About two (0.5%) patients were referred to higher dependency units, one (0.3%) was referred to the intensive care unit, while only two (0.5%) patients died (Table 3).

**Table 3:** In-hospital outcome of the study participants with dengue fever (n=374). Percentages were calculated as valid percentages excluding missing data.

In-hospital outcome	n (%)
Discharge with advice	361 (95)
Discharge on risk bond	5 (1.3)
Absconded	3 (.8)
Referred to the higher dependency unit	2 (.5)
Referred to the intensive care unit	1 (.3)
Death	2 (.5)

## Discussion

Bangladesh has experienced a marked rise in dengue incidence and related mortality over the past two decades, accompanied by wider geographic spread and increasingly severe clinical manifestations [14, 15]. This study at MMCH adds important local data from a peripheral region recently experiencing rising dengue hospitalizations.

In this study, a predominance among younger adults was reported, with over 60% of patients being in the second decade of their life. The mean age was 31.3 years. A national trend has been noticed over the past years where dengue primarily affected the younger adult population, particularly males [16, 17]. The present study also reported 315 (82.9%) of their study patients as male, pointing to a clear male predominance. Over 80% (310, 82.1%) of patients resided in urban areas, consistent with urban dengue’s vector ecology favoring the densely populated city area of Mymensingh with water stagnation after monsoon rains. A large proportion (308, 81.1%) reported recent travel to endemic areas, predominantly Dhaka. Mymensingh functions as a major administrative, economic, and educational transit hub with high socio-economic and geographic connectivity to the capital city. This proximity fuels massive, daily inter-district population exchanges. During peak transmission seasons, thousands of commuting individuals likely travel while asymptomatically or oligosymptomatically viremic, effectively introducing new viral strains into the local ecosystem. Population movement might be a key driver facilitating virus spread beyond classical urban hotspots into adjoining regions like Mymensingh. The hyper-dense population of Dhaka creates an ideal reservoir for rapid viral amplification, making it the historical epicenter of dengue in Bangladesh. However, massive and continuous population movement between the capital and neighboring divisions acts as a primary vector for viral dispersal. This high volume of human mobility effectively transports the virus from densely populated metropolitan hotspots into adjoining, expanding regional hubs like Mymensingh, facilitating geographic spread and establishing new endemic zones.

More than one-third of study patients (154, 40.5%) were overweight or obese according to Asian cut-off values. Although traditionally dengue risk was not linked to nutrition, current evidence suggests excess adiposity may exacerbate severity, potentially via inflammatory pathways and metabolic disturbances [18]. Comorbidities like diabetes mellitus (51, 13.4%) and hypertension (38, 10%) were present in a notable minority, consistent with previous literature associating chronic diseases with worsened dengue prognosis [19].

Clinically, patients predominantly presented with classic dengue manifestations; nearly all had fever, arthralgia, headache, and myalgia [20]. The mean fever duration of around seven days was comparatively longer, which indicates a longer febrile period and delayed healthcare seeking [21]. Gastrointestinal and respiratory symptoms were prevalent, with nausea (224, 58.9%), abdominal pain (155, 40.8%), cough (250, 65.8%), and chest pain (99, 26.1%). Bleeding manifestations were less frequent. This multisystem involvement and predominance of gastrointestinal symptoms have been reported in recent outbreaks in Bangladesh [14, 16, 17]. Laboratory parameters showed a low mean platelet count and a raised median ALT, which are established markers of dengue pathogenesis.

According to the revised 2009 WHO dengue class classification, 235 (62.2%) patients had at least one warning sign on admission day. But severe dengue was reported in only five (1.3%) patients. The distribution of disease severity observed at admission showed a high prevalence of warning signs alongside a very low rate of severe dengue. This provides critical insight into local healthcare-seeking behavior. Because patients were classified strictly on the day of hospital admission, this pattern indicates that the onset of warning signs functioned as the definitive clinical threshold that prompted individuals to seek tertiary care. The prolonged pre-hospitalization febrile period further demonstrates that while patients initially managed their symptoms at home during the early stages of the disease, they sought institutional admission immediately upon the manifestation of warning signs. Consequently, this behavioral pattern allowed the vast majority of the cohort to be clinically captured and hospitalized precisely at the transition into the critical phase, ensuring they received medical oversight before the illness could progress to a severe stage. Regarding in-hospital outcome, 361 (95%) were discharged with two (0.5%) deaths; outcomes were comparatively favorable vs. previous outbreaks [1, 22].

This study points toward the expansion of dengue beyond Dhaka to neighboring regions. So, a regional preparedness and vector control intensification tailored to local environments is crucial. The study findings support the epidemiological shifts of dengue across the country. Moreover, the high prevalence of recent travel to endemic areas emphasizes the role of human mobility in dengue dynamics and the need for strong surveillance.

Finally, while this study provides crucial insights into a regional outbreak, certain limitations must be acknowledged. First, as a single-center study conducted at a tertiary referral hospital, our cohort represents a selected population with a high prevalence of warning signs, which may not reflect the milder clinical presentations managed at primary or secondary care facilities. Second, due to resource constraints at a regional public facility, routine viral serotyping and secondary infection screening (such as IgG serology) were not performed, limiting our ability to correlate clinical severity at admission with specific circulating dengue serotypes or heterologous immune responses. Future multicenter longitudinal cohorts with virological assays and longer follow-up can better characterize disease evolution and sequelae.

## Conclusions

In conclusion, while hospitalized dengue patients in this regional outbreak were predominantly young adult males presenting with warning signs at admission, overall mortality remained remarkably low. Clinically, this underscores the necessity of training regional healthcare workers to recognize early warning signs immediately at triage. From a public health perspective, the shifting of dengue into peripheral divisions like Mymensingh demands a transition toward decentralized healthcare planning, regionalized resource allocation, and mobility-linked surveillance systems to manage the expanding geographical footprint of the virus across Bangladesh.

## Appendices

Appendix A 

**Table 4:** Data collection form

Name of data collector:								
Patient’s phone no:								
Emergency phone no:								
Part-1: Demographic and clinical characteristics								
Serial no:								
	Date of admission: (This is mandatory to fill)							
	Onset of fever: ______days back							
	Age (years):							
	Gender: Male Female							
	Weight (kg):							
	Height (feet/inches):							
	BMI (kg/m^2^):							
	Residence: Urban Rural							
	Monthly family income (BDT):							
	History of travel in last 30 days			Yes	No			
	If Yes in question 10, please specify the area __________________________							
	Comorbidities:							
	Peptic Ulcer			Yes		No		
	Hypertension			Yes		No		
	Diabetes Mellitus			Yes		No		
	Others: (Pls write if any other comorbidities found other than supplied options) ______________________________							
	Signs/symptoms: (On day of admission)							
	Fever		Yes					No
	Headache		Yes					No
	Myalgia		Yes					No
	Arthralgia/bone pain		Yes					No
	Palpitation		Yes					No
	Nausea		Yes					No
	Anorexia		Yes					No
	Acute vomiting (1-2 days)		Yes					No
	Persistent vomiting (>7 days)		Yes					No
	Diarrhea		Yes					No
	Cough		Yes					No
	Chest pain		Yes					No
	Respiratory distress		Yes					No
	Rash/petechiae		Yes					No
	Red eye		Yes					No
	Epistaxis		Yes					No
	Gum bleeding		Yes					No
	Hemoptysis		Yes					No
	Hematemesis		Yes					No
	Melena		Yes					No
	Hematuria		Yes					No
	Excessive menstrual bleeding		Yes					No
	Abdominal distension		Yes					No
	Abdominal pain		Yes					No
	Leg swelling		Yes					No
	Dizziness/faintness		Yes					No
	Loss of consciousness		Yes					No
	Convulsion		Yes					No
	Confusion		Yes					No
	Restlessness		Yes					No
	Jaundice		Yes					No
	Pale, cold, clammy hands and feet		Yes					No
	Edema		Yes					No
	Pleural effusion		Yes					No
	Ascites		Yes					No
	Hepatomegaly		Yes					No
	Lethargy		Yes					No
	Shock		Yes					No
	Others: ________________________							
	Features of plasma leakage: (On day of admission) (high or progressively rising hematocrit; pleural effusions or ascites; circulatory compromise or shock (tachycardia, cold and clammy extremities, capillary refill time greater than three seconds, weak or undetectable pulse, narrow pulse pressure or, in late shock, unrecordable blood pressure)			Yes			No	
	Dengue NS1 antigen: Positive Negative Test is not done							
	IgG: Positive Negative Test is not done							
	IgM: Positive Negative Test is not done							
	Blood parameters: (On day of admission) HCT (%): ________________________ Hemoglobin (g/dl): ________________________ WBC count (10^9^/L): ­­­­________________________ Platelet count (10^9^/L): ­­­­________________________							
	Liver enzymes: (On day of admission) ALT (U/L): ________________________ AST (U/L): ________________________							
	Severity of Dengue (WHO guideline 2009): (On day of admission or Day of data collection) Dengue without warning signs (Group-A) Dengue with warning signs (Group-B) Severe dengue (Group-C)	Just to remember: Warning signs: Abdominal pain or tenderness Persistent vomiting Clinical fluid accumulation Mucosal bleed Lethargy, restlessness Liver enlargement >2 cm Laboratory: increase in HCT concurrent with rapid decrease in platelet count						
	Outcome of the study: Discharge with advice/ DORB/Death/Absconded/Refer to HUD/ Refer to ICU							
